# Steroid-Induced Hypopigmentation of the Dorsal Hand: Clinicopathologic Correlation With Distinct Melan-A/SOX10 Findings

**DOI:** 10.7759/cureus.91172

**Published:** 2025-08-28

**Authors:** Veronika Skorobogatko, Svetlana Bobkova, Paul Atakpo, Igor Shendrik

**Affiliations:** 1 Radiology, Huntsville Memorial Hospital, The Woodlands, USA; 2 Biomedical Sciences, Oklahoma State University Center for Health Sciences, Tulsa, USA; 3 Dermatology, INTEGRIS Health Edmond Hospital, Edmond, USA; 4 Dermatopathology, Regional Medical Laboratory, Inc., Tulsa, USA; 5 Dermatopathology, Pathology Laboratory Associates, Inc., Tulsa, USA

**Keywords:** hypopigmentation, intralesional triamcinolone, melan-a, sox10, steroid-induced

## Abstract

Steroid-induced hypopigmentation (SIH) is an uncommon and often underappreciated complication of local corticosteroid therapy, especially in visible sites such as the dorsal hand. We report a case of a 51-year-old woman who developed an elongated hypopigmented patch following intralesional triamcinolone injection for hand pain. The lesion appeared several weeks after injection, was sharply defined, and tracked the extensor tendons without associated surface changes or significant atrophy. Histologic examination revealed a largely unremarkable epidermis with subtle atrophic changes and preserved melanocyte density as demonstrated by SOX10 staining. Notably, Melan-A staining was mostly absent or faint in occasional melanocytes, a feature we consider most characteristic of SIH and shared to some extent with idiopathic guttate hypomelanosis (IGH), but not seen in other hypopigmentary disorders such as vitiligo and postinflammatory hypopigmentation. Minimal dermal inflammation was observed, and no melanophages were present. This case emphasizes pathognomonic histologic and immunohistochemical features of SIH, in particular the characteristic Melan-A and SOX10 staining appearance in distinguishing SIH from its clinical mimickers. Underlying pathogenetic mechanisms and pertinent differential diagnoses are briefly reviewed. Considering the limited biopsy-based literature, this case adds additional histopathologic details that may improve diagnostic accuracy.

## Introduction

Steroid-induced hypopigmentation (SIH) is a recognized, though often underappreciated, complication of both topical and intralesional steroid therapy [1,2]. While most commonly encountered after high-potency topical agents or inadvertent intradermal injection [1,3], it is important to note that pigmentary changes can develop even when procedures are performed appropriately (such as intra-articular injection) [4,5]. Patients often find these changes distressing, particularly when hypopigmentation occurs in highly visible areas such as the dorsal hands or face [6]. In our experience and as described in the literature, lesions typically appear weeks to months after exposure, manifesting as sharply demarcated macules or linear, so-called “perilymphatic” streaks that may extend beyond the injection site, especially on the hands and forearms [3,4,7].

Although the clinical presentation is well documented in case reports, biopsy-based evidence remains sparse. Recent reviews have noted that less than half of reported cases include histopathologic analysis, and several authors have specifically highlighted this gap in the literature [3,6,7]. Where tissue is available for study, histologic findings are remarkably consistent: the epidermis appears essentially unremarkable, aside from a pronounced reduction of basal melanin. Melanocyte density, as demonstrated by Melan-A or SOX10 immunostaining, is generally preserved [8], and dermal inflammation is minimal or absent. Special stains such as Fontana-Masson confirm loss of pigment, while the rare electron microscopic study reveals a decrease in both the number and maturity of melanosomes, suggesting a process of functional melanogenic arrest rather than outright melanocyte loss [9].

## Case presentation

A 51-year-old right-hand-dominant woman (BMI 40.7 kg/m^2^) with multiple comorbidities, including arthritis, anxiety, asthma, and autoimmune thyroiditis, presented for evaluation of a new pale streak on the dorsum of her right hand. She recalled two recent ultrasound-guided corticosteroid injections for hand pain - 30 mg triamcinolone on January 22, 2025 (right hand), and 32 mg on April 1, 2025 (left hand) - administered while she was maintained on oral prednisone for systemic disease. About four weeks after the initial injection, she noticed a faint, slowly widening hypopigmented line that remained limited to the dorsal hand and wrist. The area of hypopigmentation was asymptomatic, and she denied unusual chemical exposures, trauma, or prior topical steroid use.

**Figure 1:**
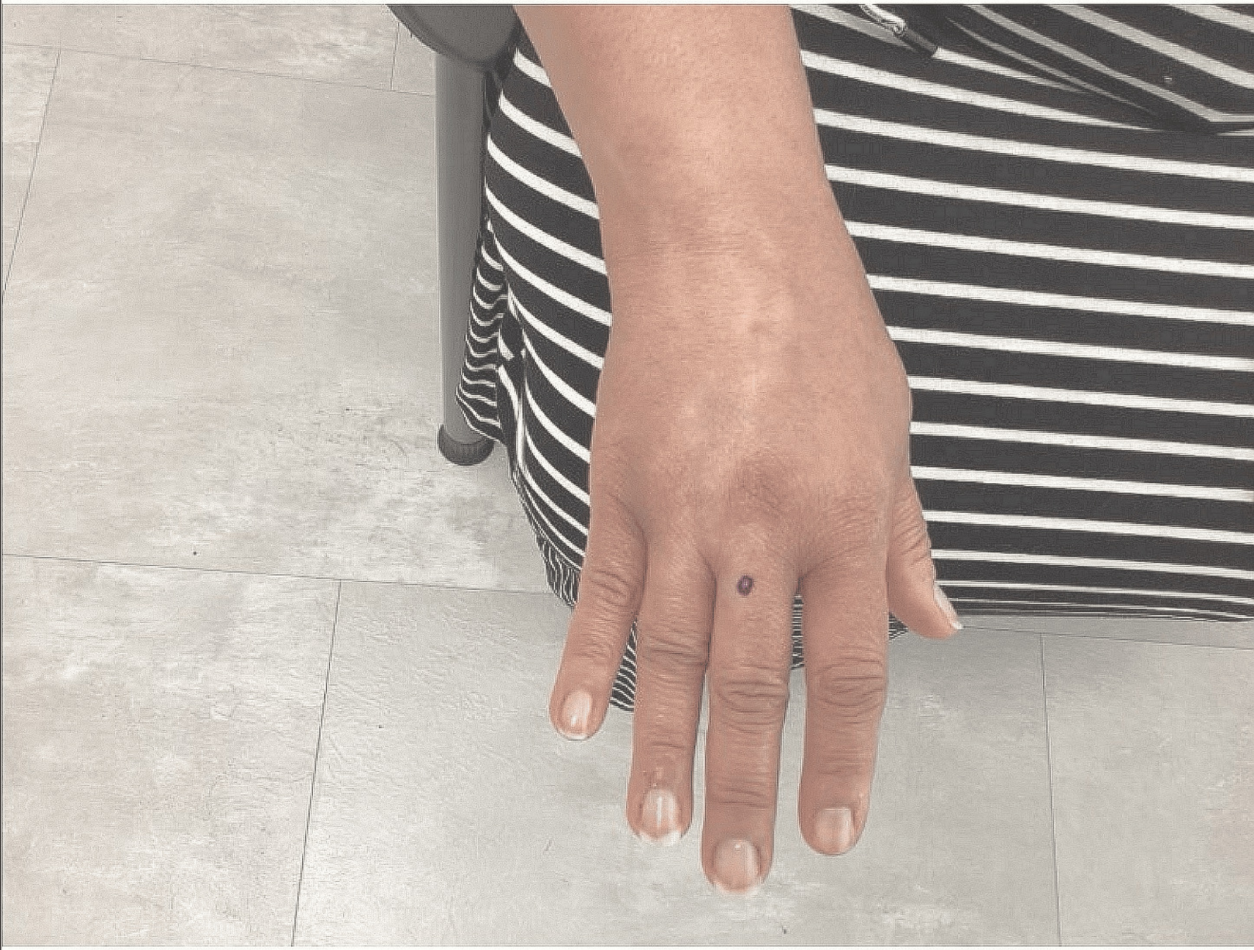
Fusiform hypopigmented patch running obliquely along the dorsal hand, centered between the second and third extensor tendons. The patch starts from the proximal phalanges of the 3rd and 4th fingers, widened to approximately 3 cm across the knuckle line, and extends over the carpus onto the dorsal wrist, where it slowly tapers in the direction of the distal forearm. Its radial and ulnar borders followed the contours of the extensor tendons, blending imperceptibly into normally pigmented skin. A punch biopsy site is centrally located within the hypopigmented patch on the dorsal proximal phalanx of the middle finger.

On examination (Figure 1), an elongated, fusiform patch of hypopigmentation following the extensor tendons was noted. The skin surface was otherwise unremarkable - flat, supple, and without scale or atrophy. Sensation, capillary refill, and sweating were intact. The clinical appearance, distribution, and timing, taking together with the patient’s corticosteroid exposure, raised suspicion for steroid-induced hypopigmentation, although other causes such as early vitiligo were considered, especially in light of the concurrent autoimmune conditions. Wood’s lamp examination revealed an ivory rather than chalk-white hue, favoring reduced melanin content over true melanocyte loss.

Given the patient’s concerns, a punch biopsy was performed to exclude vitiligo or interface dermatitis.

**Figure 2:**
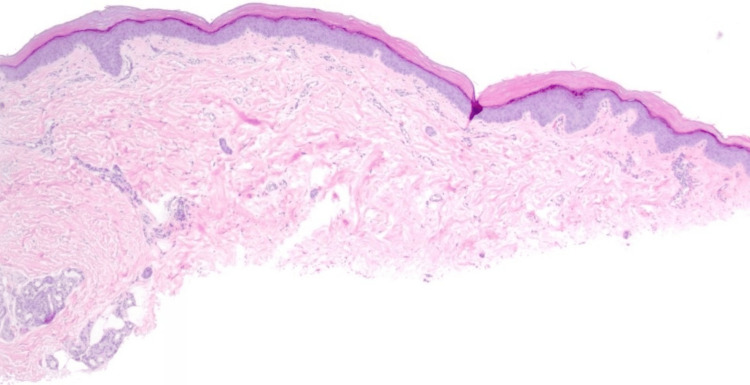
Largely unremarkable epidermis, with slight simplification of the rete ridges, minimal pigmentation of the basal keratinocytes. No interface changes, melanophages, or inflammation. H&E x20

Shave biopsy sections (Figure 2) demonstrated a largely unremarkable epidermis, with minor atrophy and slight simplification of the rete ridges. The basal keratinocytes show uniform minimal pigmentation, though quantitative assessment is limited by the lack of adjacent normal skin. The dermal-epidermal junction demonstrates no significant interface changes, and there is no lichenoid inflammation. The reticular dermis was notable for mild collagen compaction and only rare chronic inflammatory cells, with no eosinophils or histiocytes. No melanophages were seen, supporting a non-inflammatory pigment loss. Eccrine structures were present in the deep dermis and subcutis without evidence of compression or distortion.

**Figure 3:**
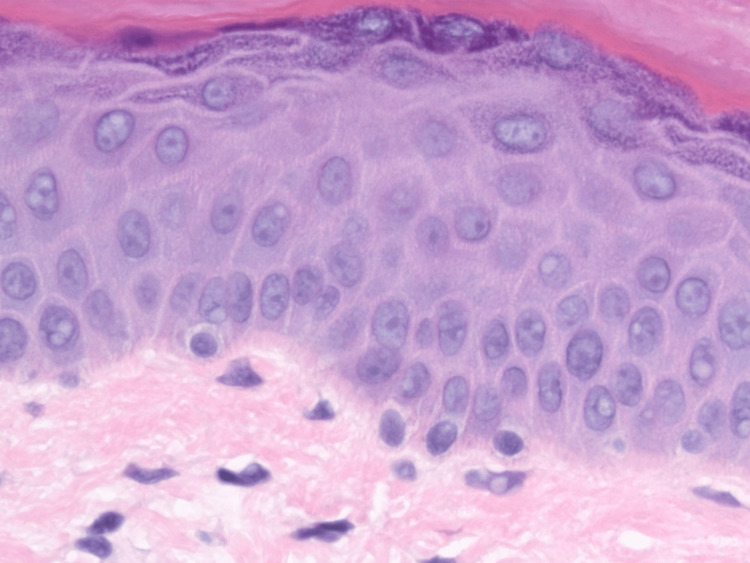
Rare solitary basally situated melanocytes of usual size with thin cytoplasmic rim (H&E x200)

Higher-power evaluation (Figure 3) demonstrated epidermis where keratinocytes exhibited uniform, round-to-oval nuclei with finely granular chromatin and inconspicuous nucleoli; the cytoplasm was pale eosinophilic without vacuolar change. Along the dermo-epidermal junction, the melanocytes were identifiable only as occasional solitary cells situated between basal keratinocytes. The melanocytes were discrete with nuclei of usual size (4-6 micrometers), equal or slightly smaller than the nuclei of adjacent basal keratinocytes. The melanocytes showed round nuclei, smooth nuclear contours, and delicate, dusty chromatin; nucleoli were absent or pinpoint. The cytoplasmic rim was extremely thin and lightly basophilic, lacking the coarse brown melanin granules normally imparting amphophilia, and dendritic projections were either rudimentary or not appreciable. There was no confluence, nesting, suprabasal ascent, pagetoid spread, or cytologic atypia. The papillary dermis was essentially unremarkable.

**Figure 4:**
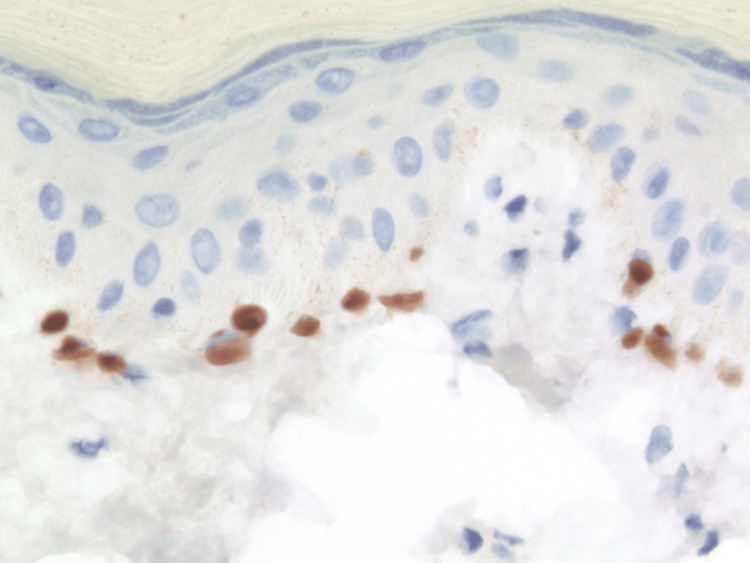
Double immunohistochemical staining for Melan-A and SOX10 (original magnification ×20). Junctional melanocytes exhibit SOX10-positive (brown) nuclear staining with minimal to absent Melan-A cytoplasmic staining (red chromogen). Focal cytoplasmic Melan-A positivity is observed in a melanocyte at the right corner of the image.

Double immunohistochemical stain with Melan-A/SOX10 (Figure 4) and Fontana-Masson special stain (not shown) was performed. The dermo-epidermal junction was populated by well-spaced melanocytes that stood out as compact ovoid to elongate nuclei exhibiting crisp brown SOX10 positivity. There was a striking reduction in Melan-A staining of melanocytic cytoplasm, with absent or only faint positivity present in rare melanocytes (controls were stained appropriately). The decreased intensity of the red Melan-A stain underscored a reduction in melanosome load and impaired pigment synthesis/transfer.

It is worth noting that a uniformly distributed, dust-like intraepidermal pigment, discernible on the double Melan-A/SOX10 stain, was also highlighted by the Fontana-Masson stain, supporting the melanocytic nature of these particles. These findings support reduction, but not absolute absence, of dermal pigment correlating to the clinical impression. Immunohistochemical stain with PRAME was additionally performed for completeness of evaluation and was, as expected, entirely negative.

## Discussion

Steroid-induced cutaneous hypopigmentation (SIH) represents an uncommon but well‑characterized adverse event following local corticosteroid administration, first recognized in the early 1960s with the advent of depot steroid formulations [10]. Goldman’s seminal 1962 report described cutaneous reactions after intralesional corticosteroids [10], and Schetman and colleagues soon thereafter established the characteristic association between triamcinolone deposition and hypopigmentation accompanied by localized atrophy [11]. These foundational observations linked clinical hypopigmentation to the physical presence of insoluble steroid micro‑crystals within the dermis, a concept reshaped and expanded in the 1980s by Friedman et al. through the introduction of the terms “perilesional” and “perilymphatic” linear hypopigmentation [12] to describe serpiginous pigment loss following superficial lymphatic channels after intralesional therapy. Contemporary literature has refined these descriptors further, coining evocative terms like “corticosteroid snow” and “streaky leukoderma” to reflect the characteristic morphologic patterns seen clinically [13].

Although the true incidence of steroid‑induced pigment alteration is low, it is clinically relevant. Early rheumatology trials from the 1960s noted approximately a 3 % rate of pigmentary changes after local triamcinolone injections [10], whereas modern dermatologic audits suggest cutaneous atrophy and dyspigmentation occur in roughly 0.5 % of intralesional injections overall [14]. A pediatric musculoskeletal cohort described a somewhat higher prevalence (1.8 %) of hypopigmentation following intra‑articular triamcinolone administration [15], highlighting variability depending on patient population and injection‑site anatomy.

Risk factors identified include the use of large‑particle corticosteroid preparations-such as triamcinolone acetonide at concentrations of 40 mg/mL-superficial injection technique, volumes exceeding 0.5 mL per site, and administration at richly lymphatic sites like the dorsal hand and forefoot [12, 15, 16]. Furthermore, topical ultrapotent fluorinated steroids may induce pigmentary loss, particularly in darker phototypes or sites with thin epidermis and occlusion [16]. This cumulative clinical and pharmacologic risk profile underscores the multifactorial nature of steroid‑induced hypopigmentation risk and the importance of cautious administration practices and pre‑injection counseling [16, 17].

Clinically, SIH typically manifests as ivory macules or linear chalky streaks appearing 2 to 6 weeks post‑exposure, often extending several centimeters beyond the injection track itself, reflecting lymphatic or tendon alignment [12, 13]. Histologically, melanocyte numbers are preserved, but basal melanin content is diminished, pointing towards a functional melanocyte impairment rather than outright cytotoxic loss [16]. Most cases demonstrate spontaneous repigmentation within 3-12 months, although persistence beyond two years and rare irreversible sequelae have been documented [14]. Adjunctive interventions such as excimer light therapy, narrow‑band UV‑B phototherapy, fractional CO₂ laser, and serial saline injections (“saline rechallenge”) have been reported to accelerate repigmentation or mitigate cosmetic concerns [14, 15]. Nonetheless, the optimal therapeutic approach remains individualized, informed by lesion location, patient preference, and pigment loss severity [14, 15].

While SIH is clinically distinctive, it shares some morphologic and histopathologic overlap with other hypopigmentary disorders such as vitiligo, idiopathic guttate hypomelanosis (IGH), post‑inflammatory hypopigmentation (PIH), and progressive macular hypomelanosis (PMH). Distinguishing these entities is critical, as their pathogeneses, prognoses, and treatments differ substantially.

The clinical morphology and distribution of these disorders are summarized in Table 1 below, highlighting key distinctions:

**Table 1:** The clinical morphology and distribution of hypopigmented disorders ^*^[12,13,16] SIH: steroid-induced hypopigmentation; IGH: idiopathic guttate hypomelanosis; PIH: post-inflammatory hypopigmentation; PMH: progressive macular hypomelanosis.

Parameter	Steroid-induced hypopigmentation (SIH)*	Early vitiligo	Idiopathic guttate hypomelanosis (IGH)	Post-inflammatory hypopigmentation (PIH)	Progressive macular hypomelanosis (PMH)
Clinical morphology & distribution	Linear/serpiginous patch along lymphatics or tendon lines adjacent to steroid depot; subtle lipo-atrophy; onset 2–6 weeks post-injection	Sharply demarcated chalk-white macules/patches; acral or periorificial; Koebnerisation common	Multiple 2–6 mm round/oval macules on sun-exposed limbs; increasing with age	Irregular hypopigmented areas at prior eczema, trauma, scars; hazy borders	Ill-defined nummular macules on trunk, midline confluence; rare on limbs

SIH lesions exhibit a linear or serpiginous pattern distributed along lymphatic vessels or tendon sheaths, as demonstrated in our patient’s dorsal hand leukoderma extending along the extensor tendon. This is a direct consequence of the perilymphatic migration of insoluble steroid crystals post‑injection [12, 13]. Early vitiligo lesions show more sharply demarcated chalk‑white patches that fluoresce brightly under Wood’s lamp due to total melanin absence and loss of melanocytes. In contrast, SIH presents ivory or off‑white pigmentation with less intense Wood’s lamp accentuation, consistent with partial melanin depletion and preserved melanocyte populations [16]. IGH typically manifests as multiple small, scattered macules on sun‑exposed limbs with a characteristic “skip area” pattern on melanin staining. PIH often follows inflammatory dermatoses and features irregular, hazily bordered areas with pigmentary incontinence. PMH involves ill‑defined macules predominantly on the trunk with red follicular fluorescence attributable to Cutibacterium acnes colonization.

Histologic evaluation plays a crucial role in resolving the clinical differential diagnosis. It is reported that SIH is characterized by mild epidermal thinning, blunted rete ridges, diminished basal melanin on Fontana‑Masson stain, and preserved melanocyte density positive for Melan‑A and SOX10, albeit with small, dendrite‑poor “shrunken” melanocytes [16]. Vitiligo shows complete melanocyte loss with no melanin granules. IGH displays flattened epidermis, thick basket‑weave stratum corneum, and “skip areas” of melanin loss and reduced but not absent melanocytes. The melanocytes in IGH typically exhibit enlarged cell bodies but have markedly reduced, retracted or absent dendritic processes (not that cytologically dissimilar to SIH). This dendritic diminution impairs melanin transfer to keratinocytes, contributing to the characteristic hypopigmentation despite the presence of melanocytes. Ultrastructural studies confirm these dendritic abnormalities alongside signs of melanocyte senescence and decreased melanosome numbers. PMH maintains near‑normal melanocyte counts and architecture, including preservation of melanocytic dendrites; however, ultrastructural analyses consistently reveal alterations in melanosomes with reduced total melanin content and a predominance of small, aggregated, membrane‑bound melanosomes at earlier maturation stages (I-III), as opposed to the larger, mature (stage IV) single melanosomes found in healthy skin. These clustered, less‑developed melanosomes accumulate within the basal and suprabasal keratinocytes, resulting in diminished visible pigmentation despite normal melanocyte numbers and cytologic appearance. Therefore, the main defect in PMH is tied to altered maturation and packaging of melanosomes inside melanocytes and keratinocytes.

Our patient’s biopsy revealed the diagnostic combination of preserved SOX10+ nuclei and a faint or visually absent Melan‑A rim around melanocyte cytoplasm. While the absence of normal skin for comparison limits quantification of Fontana‑Masson staining, it shows no “skip” areas and appears diminished, consistent with steroid‑induced functional melanocytostasis. Absence of interface dermatitis or lymphocytic infiltrate excludes post‑inflammatory types.

The pathogenesis of SIH is complex and involves the convergence of several pathologic axes, summarized in Table 2.

**Table 2:** : Pathophysiological mechanisms of steroid-associated hypopigmentation α-MSH: alpha-melanocyte-stimulating hormone; TRP-1: tyrosinase-related protein 1; MITF: microphthalmia-associated transcription factor; TNF-α: tumor necrosis factor alpha; IL-1: interleukin 1; IGH: idiopathic guttate hypomelanosis; SIH: steroid-induced hypopigmentation

Mechanism	Supporting Evidence	Expected Histology/Clinical Footprint
Dermal vasoconstriction & “blanching”	Solomon et al. demonstrated rapid constriction of dermal arterioles by topical/injectable corticosteroids, mimicking adrenaline effects; impaired blood flow starves melanocytes of oxygen and α-MSH signals needed for melanogenesis [17].	Patchy basal pallor; melanocytes present but “shrunken”; hypopigmentation mirrors vascular distribution
Direct suppression of melanogenic enzymes	In vitro studies show glucocorticoids down-regulate tyrosinase, TRP-1, and mitf transcripts; reduced melanogenic enzyme production limits melanin synthesis [14].	Diffuse decrease in melanin granules; melanocytes intact but cytoplasm diminished
Melanocyte energy crisis/ cytotoxicity	High local steroid concentration induces oxidative stress, mitochondrial swelling, cytoplasmic vacuolization; ultrastructure reveals swollen early melanosomes and reduced dendricity, analogous to IGH ultrastructure.	Epidermal thinning; condensed melanocyte nuclei; some necrotic melanocytes
Crystal dispersion along lymphatics (“corticosteroid snow”)	Microcrystalline steroid deposits embolize lymphatics, producing linear serpiginous patterns beyond injection site; dye migration studies support lymphatic spread [12,13].	Linear streaks of hypopigmentation, often overlying lipoatrophy
Immune modulation/anti-cytokine effects	Steroids suppress IL-1, TNF-α, prostaglandins, and leukotrienes, which normally promote melanogenic transcription factors; mouse models lacking these mediators show hypopigmentation [16].	Lack of interface change or lymphocytic infiltrate distinguishes SIH from inflammatory hypopigmentation

Our patient’s potent triamcinolone acetate depot injections provided a high concentration of insoluble corticosteroid microcrystals that, placed superficially over the dorsal hand with its thin dermis and rich lymphatic network, predisposed to crystal dispersion and perilymphatic hypopigmentation [12, 13]. The concurrent use of systemic prednisone (10 mg daily) likely augmented local vasoconstriction and prolonged melanocytic enzymatic suppression [16, 17], thereby delaying repigmentation. Additionally, morbid obesity can impair lymphatic clearance via adipose‑tissue-related cytokines (e.g., leptin) and microvascular dysfunction, prolonging steroid retention and tissue toxicity. The dorsal hand’s relatively low follicular density-an important reservoir for melanocyte stem cells-further impedes repopulation compared with the scalp or trunk. Thus, the interplay of depot burden, systemic steroid exposure, patient physiology, and site‑specific factors exacerbates the pathogenic mechanisms affecting melanogenesis.

Understanding SIH as a manifestation of reversible melanocytic dysfunction rather than permanent cell loss bears crucial clinical relevance. The presence of preserved but dormant melanocytes predicts a favorable prognosis for spontaneous repigmentation, typically occurring within 6-12 months as corticosteroid depot levels wane [16]. Photostimulatory treatments, including excimer 308‑nm laser and narrow‑band UV‑B phototherapy [15], may accelerate melanin production and pigment recovery. Additionally, fractionated CO₂ laser and serial intradermal isotonic saline injection have demonstrated benefit in alleviating steroid‑induced atrophy and associated pigmentary changes [14, 15]. Importantly, recognizing distinct pathogeneses directs treatment choice and patient counseling: whereas SIH responds well to phototherapy and mechanical interventions [14, 15], early vitiligo warrants immunomodulatory therapies. IGH is generally recalcitrant to light‑based therapies, and PMH treatment focuses on antimicrobial strategies targeting Cutibacterium acnesalongside phototherapy.

Our patient’s constellation of risk factors-including depot load, systemic prednisone, obesity, and hand anatomy-likely prolonged repigmentation time. The documented histologic diagnosis facilitates clinical follow‑up, diminishes the patient’s underlying anxiety, and supports reassurance regarding prognosis. She was counseled that corticosteroid‑related pigment loss is usually reversible within 6-12 months. She elected observation and will consider adjunctive narrow‑band UV‑B therapy if repigmentation is not evident at three‑month follow‑up.

## Conclusions

We present a well-documented case of steroid-induced hypopigmentation (SIH) supported by histologic and immunohistochemical analysis, highlighting the distinctive pattern of dual Melan-A and SOX10 staining characterized by a near absence of cytoplasmic Melan-A positivity alongside preserved nuclear SOX10 expression, with maintained melanocyte density and distribution. While a similar “shrunken-cell” melanocyte phenotype may occasionally be observed in idiopathic guttate hypomelanosis (IGH), differentiation is achievable through distinct clinical appearance and presence of uniform (SIH) versus patchy (IGH) melanin retention on Fontana-Masson staining. Our review of the underlying pathogenetic mechanisms emphasizes the multifactorial nature of SIH, integrating vasoconstriction, enzymatic suppression, and lymphatic crystal dissemination. Recognition of these features is critical for accurate diagnosis and guiding appropriate management strategies in patients presenting with hypopigmented lesions following corticosteroid therapy.
